# Macroscopic liquid-state molecular hydrodynamics

**DOI:** 10.1038/srep41658

**Published:** 2017-01-31

**Authors:** R. G. Keanini, Peter T. Tkacik, Eric Fleischhauer, Hossein Shahinian, Jodie Sholar, Farzad Azimi, Brid Mullany

**Affiliations:** 1 The University of North Carolina at Charlotte, Department of Mechanical Engineering and Engineering Science, Charlotte, NC, 28223, USA

## Abstract

Experimental evidence and theoretical modeling suggest that piles of confined, high-restitution grains, subject to low-amplitude vibration, can serve as experimentally-accessible analogs for studying a range of liquid-state molecular hydrodynamic processes. Experiments expose single-grain and multiple-grain, collective dynamic features that mimic those either observed or predicted in molecular-scale, liquid state systems, including: (i) near-collision-time-scale hydrodynamic organization of single-molecule dynamics, (ii) nonequilibrium, long-time-scale excitation of collective/hydrodynamic modes, and (iii) long-time-scale emergence of continuum, viscous flow. In order to connect directly observable macroscale granular dynamics to inaccessible and/or indirectly measured molecular hydrodynamic processes, we recast traditional *microscale* equilibrium and nonequilibrium statistical mechanics for *dense, interacting* microscale systems into self-consistent, *macroscale* form. The proposed macroscopic models, which appear to be new with respect to granular physics, and which differ significantly from traditional kinetic-theory-based, macroscale statistical mechanics models, are used to rigorously derive the continuum equations governing viscous, liquid-like granular flow. The models allow physically-consistent interpretation and prediction of observed equilibrium and non-equilibrium, single-grain, and collective, multiple-grain dynamics.

Molecular hydrodynamics typically uses two approaches to study molecular-scale dynamics in liquids and gases, the first measuring light^1^, neutrons^2^, or high-frequency sound scattered from an interrogation volume^3^, the second computationally simulating the dynamics of spatially-limited, N-body systems subject to various forcing mechanisms, and interacting through specified interparticle potentials^3^,^4^,^5^,^6^,^7^.

Analog systems, including charged colloids^3^ and solutions of large macromolecules^1^,^8^,^9^, have also been used in studies of liquid-state molecular-scale dynamics. In contrast to molecular light scattering experiments, this approach often allows direct observation of single-particle dynamics and correspondingly, validation of molecular dynamic (MD) single-particle simulations. Vibrated grain systems hold unrealized potential as macroscopic analogs for investigating molecular hydrodynamic processes. Striking similarities have recently been observed, for example, between the macroscale, steady-state structure of vibrationally excited granular media and the equilibrium structure of molecular liquids^10^. These and earlier studies^11^,^12^ have likewise reported ‘particle-in-a-cage’ single-grain dynamics, similar to a well-known single-molecule effect predicted (via MD simulation) in dense, interacting molecular fluids^3^,^4^. [Aside: Throughout this paper, ‘dense fluids’ will refer to, and be used interchangeably with, ‘liquids’ and ‘dense, interacting gases’. We will assume a liquid state when the grain velocity autocorrelation function, *ψ*(*t*), becomes negative and then re-approaches zero at large times, a dynamical indicator of trapped-but-not-solidified particle dynamics^3^. Likewise, ‘molecule’, ‘atom’ and ‘particle’ will be used synonymously. An ‘interacting fluid’ is one having high enough density that interparticle interactions - e.g., Coulomb and van der Waal forces in molecular liquids, elastic and frictional contact forces in granular liquids - exert a significant influence on single-particle and collective dynamics].

This paper proposes that confined piles of high-restitution grains subject to low-amplitude vibration can serve as macroscopic analogs for qualitative investigations of molecular hydrodynamic processes. In order to use an analog to predict, interpret, or expose physical processes in liquid-state molecular systems, two broad correspondences, one experimental and one theoretical, and both holding over a wide range of single-particle and multiple-particle dynamic conditions, must be established. Experimentally, the macroscopic system must, at minimum, exhibit: (i) a sustained tendency toward local, *macroscale* statistical mechanical equilibrium, and (ii) small, linear nonequilibrium departures from equilibrium that qualitatively mimic (weak) nonequilibrium fluctuations observed in molecular hydrodynamic systems. Theoretically: (i) classical microscale models describing the equilibrium and weakly-nonequilibrium statistical mechanics of dense, interacting N-particle systems must be recast in self-consistent macroscale form, and (ii) resulting macroscale models must reliably predict single- and multiple-particle dynamics, as observed from short, particle-collision-time-scales to long, continuum-flow-time-scales.

With regard to local macroscale equilibrium states, the granular physics community has long-maintained the opinion that dynamic, flowing grain systems are intrinsically nonequilibrium^10^,^11^,^13^. In terms of the microscale statistical mechanics of in-grain molecular processes, this is correct. However, as shown in this article, the random, *macroscale* dynamics of a large family of high-restitution grains, undergoing low-amplitude vibration, *when interpreted from the vantage point of a self*-*consistent macroscale version of Gibbs ensemble*-*based, microscale equilibrium statistical mechanics model*^14^,^15^, are wholly consistent with existence of local, *macroscale* statistical mechanical equilibrium. This view is buttressed by recent observations of nominal Maxwell-Boltzmann (MB) velocity distributions and microscale-like radial distribution functions, *g*(*r*), in vertically-vibrated, high-restitution grain columns^10^. [Note, Shattuck *et al*.^10^ observed nominal MB distributions for grain velocities not exceeding twice the rms value]. These were interpreted as corresponding to nonequilibrium, (microscale) steady state processes^10^; from the vantage point of macroscale equilibrium statistical mechanics, however, these are manifestations of grain-scale, *equilibrium* dynamic processes.

Considering the use of vibrated grain analogs for studying weak nonequilibrium fluctuations in molecular systems, molecular scattering experiments and MD simulations have exposed a multiplicity of single-particle and collective dynamic features, extant over short, sub-collision to long hydrodynamic time-scales^1^,^3^. By contrast, the limited set of measurements reported here represents a small sampling of the (macroscale) dynamical space available to our vibrated grain system. Nevertheless, the following observations, detailed below, and each qualitatively mimicking well-characterized nonequilibrium dynamic behaviors in molecular liquids, provide compelling evidence that confined piles of high-restitution grains, undergoing low-amplitude vibration, likely share other essential molecular-scale dynamical properties: (i) near-collision-time-scale, hydrodynamic organization of single-grain dynamics, (ii) collective, vibrationally-excited (viscously damped) hydrodynamic acoustic modes that propagate - at surprisingly low speeds - throughout the experimental grain pile, (iii) apparent hydrodynamic diffusive vortical modes (typically called transverse momentum modes), which together with the acoustic modes in (ii), induce (iv) a slow, steady, laminar viscous grain flow - which is predictable via the Navier-Stokes equations - throughout our vibrating grain pile.

Development of a theoretical framework for studying liquid-like grain flows must, on one hand, realistically describe the macroscale dynamics of N-grain systems, ideally over the entire range of experimentally-accessible macroscopic length and time-scales, and on the other, align conceptually and mathematically with traditional microscale equilibrium and nonequilibrium statistical mechanics models of interacting liquid systems. Here, we: (i) recast the traditional^16^, ensemble-based, microscale model of local equilibrium within interacting systems into macroscale form, (ii) emphasize the applicability of generalized (memory-dependent) Langevin models for describing macroscale departures from local macroscale equilibrium, and (iii) use a well-known microscale argument to rigorously derive nonequilibrium, macroscale, hydrodynamic conservation laws.

In closing this Introduction, we highlight the essential difference between the new theoretical approach proposed here and traditional kinetic-theory-based methods used in previous grain flow studies^13^,^17^. Kinetic models require modeling of intergranular collisions; in dense, interacting systems this remains an unresolved challenge, manifesting itself most apparently in unreliable transport property predictions^13^,^17^. By contrast, combined application of the Gibbs equilibrium model^14^,^15^ and any of several models of linear, nonequilibrium fluctuation^3^,^6^,^18^,^19^,^20^ circumvents collision modeling. Two important points are noted. (i) Operationally, linear nonequilibrium models of dense fluids focus attention on a wide variety of correlation and system response functions, the evolution or equilibrium character of which are tightly constrained by particle- and hydrodynamic-scale conservation laws, fluctuation-dissipation theorems, Kubo relations, thermodynamic sum rules, and equilibrium thermodynamic relationships. This means that theoretical predictions of, e.g., continuum grain flows and associated transport properties, are likewise tightly constrained, and thus likely more robust than those generated via kinetic models. (ii) Physically, the Gibbs-equilibrium/linear-nonequilibrium framework applies only if the many-body system under study behaves in ways that are consistent with, and are predictable by, the framework. As noted by Kubo^21^, many, and likely most, many-body problems have no underlying macroscale equilibrium state, or are subject to large, nonlinear excursions from equilibrium.

## Results

This paper reports a set of experimental and theoretical contributions to the study of dense, liquid-like, vibration-driven granular systems. Based on these, we propose an experimentally-accessible analog approach for studying molecular hydrodynamic systems. As a guide to what is presented, both in the paper and in the Supplementary material, we highlight the main results:

1. We show experimentally that a large family of vibrated, high-restitution grains exist in local statistical mechanical equilibrium; see subsection entitled ‘Macroscale Equilibrium Statistical Mechanics’ below. This observation, along with a scaling argument (Supplement 1), supports our contention that the peculiar (i.e., random), collision-time-scale dynamics of N-grain systems are nominally Hamiltonian (non-dissipative). This observation is also consistent with the assumption that collision-time-scale grain momenta are independent of inter-grain (elastic) potentials and vice versa, analogous to the picture generally assumed in dense molecular-scale systems.
2. In analogy with single-particle dynamics predicted in dense gases^3^, we observe near-collision-time-scale, hydrodynamically-induced organization of single-grain dynamics; see ‘Hydrodynamic Organization of Short-Time-Scale Single-Grain Dynamics’ below. In contrast to microscale theoretical predictions, however, we observe hydrodynamic organization (i.e., organization produced by continuum-scale, collective dynamics) on sub- and near-collision time-scales; MD models predict this effect on slightly longer time-scales. In addition, the hydrodynamic feature that produces short-time-scale organization here arises from diffusion of linear momentum, injected into (all points within) the grain pile by vibration; see Supplement 2.
3. In conformity with theoretical arguments applied to single atoms and molecules^3^, we observe that sub- and near-collision-time-scale, single-grain dynamics are consistent with generalized Langevin dynamics. This feature becomes apparent in the short-time, non-exponential (algebraic) decay of the single-grain autocorrelation function, and can only be explained using a memory-dependent (generalized) Langevin equation; see ‘Hydrodynamic Organization of Short-Time-Scale Single-Grain Dynamics’ below. Likewise, on long time-scales (specifically, time-scales long relative to fast elastic modes excited within individual grains), individual grains exist in a discontinuous bath of small, high-frequency elastic bumps excited on the surface of each grain. Dynamically, these virtual bath particles produce ordinary (memory-free), single-grain Brownian motion.
4. Similar to atomic liquids and dense gases exposed to weak external perturbations, the low-frequency, collective response of vibrated grain piles is characterized by excitation of hydrodynamic modes. As described in ‘Hydrodynamic Modes’ below, for example, we observe viscously damped acoustic modes propagating throughout the grain pile. While not detailed in the paper, our analyses also indicate existence of a weak diffusive entropy mode, as well as (two) uncoupled (diffusive) vorticity modes; the first is consistent with experimental observations in dense fluids^1^,^3^,^6^, and the second with well-known theoretical predictions^3^,^6^,^22^.
5. Exact molecular- and grain-scale, i.e., discrete-particle versions of mass, momentum, and energy conservation, can be restated - via a little-used coarse-graining procedure appropriate to dense, interacting systems^6^,^7^ - in continuum form; see Supplement 2. Introduction of empirical constitutive relations, tying local (ensemble averaged) mass, momentum, and energy fluxes to local gradients in equilibrium thermodynamic variables, as well as gradients in local (bulk) dynamical variables, e.g., the local bulk velocity, then leads to the Navier-Stokes equations (which govern continuum-scale transport). This paper adapts this theoretical framework to derive self-consistent continuum mass, momentum, and energy transport equations appropriate to vibrated grain systems; see Supplements 1 and 2.
6. We describe two rough consistency checks on the proposed theoretical framework: (i) A macroscale Green-Kubo relation^3^,^6^,^20^ for the effective grain-fluid dynamic viscosity is used to obtain rough estimates of the effective kinematic viscosities of the nine grains used in our experiments; see Supplement 2. (ii) A simplified model of vibration-driven flow of a Newtonian fluid within a hemisphere, subjected to the same multimodal vibration used in our experiments, is used to predict the continuum flow of grains within our experimental system; see ‘Time-Averaged Grain Flow’ below and Supplement 2. As detailed in Supplement 2, predicted kinematic viscosities are, for seven of the nine grains tested, quantitatively consistent with those we have experimentally measured. Likewise, as shown in ‘Time-Averaged Flow’ below, predicted grain flow fields are qualitatively consistent with the non-trivial, helical grain flow fields we have observed.
7. Given evidence that high-restitution grains behave singly and collectively, on short sub-collision to long continuum time-scales, in ways that are qualitatively consistent with the dynamic behaviors observed in dense, interacting, molecular hydrodynamic systems, and given that this array of macroscale dynamic behaviors can be consistently interpreted and predicted using macroscale versions of traditional microscale equilibrium and nonequilibrium statistical mechanical models, we propose that systems of high-restitution grains can serve as analogs for studying dense, interacting molecular hydrodynamic systems.

## Experiments and Macroscale Equilibrium and Nonequilibrium Statistical Mechanics Models

### Experimental System

**Figure 1:**
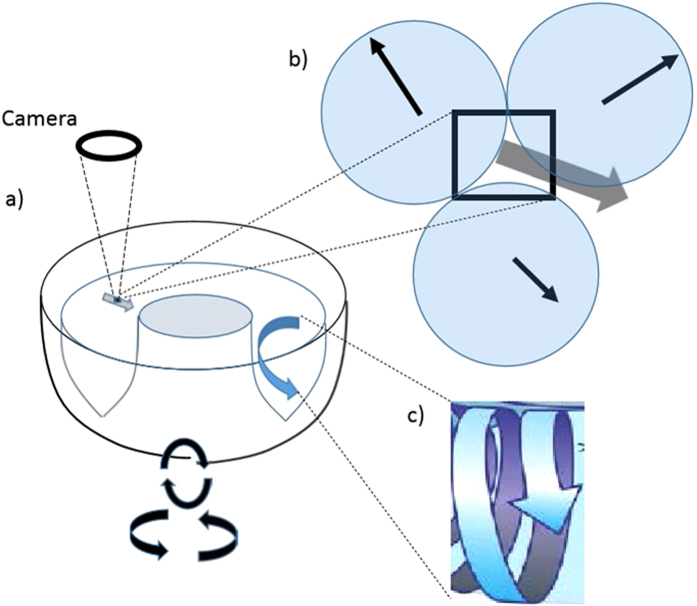
Experimental measurement of grain dynamics. (**a**) Grain piles confined to a commercial vibrational finishing bowl undergo three simultaneous modes of single-frequency (29.3 Hz) vibration (see text). Local (interrogation area = 4 mm × 4 mm) and large field-of-view (LFOV, 150 mm × 200 mm) images of grain motion are captured at 500 frames/s at the surface of the vibrating pile; corresponding two-dimensional local and LFOV velocities are determined via PIV. (**b**) The nine grains tested have characteristic dimensions ranging from approximately 10 to 35 mm. Local PIV interrogation area shown as a bold square; local time-average grain (fluid) velocity, **v**, shown in grey and local instantaneous random (peculiar) grain velocities relative to **v** shown in black. (**c**) Time-average grain flows are, for all grains tested, helical.

Our experimental system, described in detail in ref. 23, consists of a commercial vibrational finishing bowl, driven in three simultaneous, single-frequency modes of vibration: circular-in-a-vertical-plane, precession-about-a-vertical-axis, and horizontal-back-and-forth-twisting. The bowl can be loaded to a maximum depth of about 0.3 m, with various, typically centimeter-scale grain media. A high-speed video camera captures grain motion either over large portions of the grain pile surface, or over small (4 mm × 4 mm), single-grain-scale interrogation areas on the surface. Image data is fed to a particle imaging velocimetry (PIV) system which allows processing of captured images, observed over time-scales ranging from 2 ms to 10 s. See Fig. 1. Further details are described below and in ref. 23. In the following, the grain *collision time*-*scale, τ*_*c*_, corresponds to the inverse driving frequency, 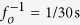, used to vibrate the bowl and grain pile.

### Macroscale Equilibrium Statistical Mechanics

Macroscale equilibrium and nonequilibrium statistical mechanical models, applied to the peculiar (random) dynamics of high-restitution N-grain systems undergoing low-amplitude vibration, are derived in Supplement 1. The equilibrium model is based on three assumptions: (i) On short, grain-collision-time-scales, the dynamics of any N-grain system are Hamiltonian, i.e., non-dissipative. In addition, the system kinetic energy is assumed to depend only on the squared linear momentum of each grain in the system, while the potential energy, determined by elastic contact between grains, is assumed independent of grain momenta. (ii) For any given system of N grains within a confined, high-restitution pile of grains, each grain characterized by a given shape, mass density, and a set of elastic material properties, and with the pile subjected to any combination of low amplitude vibrational forcing, a well-defined set of macroscale, athermal, purely mechanical energy states exists. (iii) The fundamental postulate of equal *a priori* probability is assumed, i.e., at any instant, the probability of observing the N-grain system in one of its available macroscale energy states is uniform. Given these assumptions, and using standard arguments^16^,^24^, bridge relationships connecting the discrete, random, Hamiltonian, vibration-driven dynamics of N-grain systems to a complete, self-consistent set of multiple-grain-scale equilibrium thermodynamic state variables, including entropy, internal energy, temperature, and pressure, can be derived; see Supplement 1.

**Figure 2:**
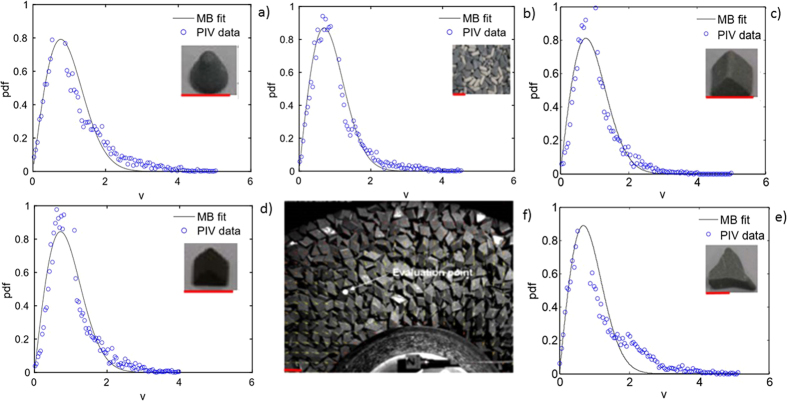
Experimental signature of local statistical mechanical equilibrium. Data points show distributions of horizontal peculiar (random) grain speeds, as measured at the point shown in (**f**). Curve fits assume two-dimensional Maxwell-Boltzmann (MB) speed distributions and implicitly assume that unmeasured vertical (peculiar) grain velocities are Gaussian, independent of horizontal velocities, and have the same variance measured in the horizontal plane. Simultaneous measurements of mutually orthogonal horizontal, peculiar (random) velocity components show that each component, for all grains tested, are well-fit by Gaussian pdf’s; this observation underlies the assumption that the vertical component is likewise Gaussian. Speeds, *v*, and probability density functions are in units of *cm* *s*^−1^ and *s* *cm*^−1^, respectively, and the red scales represent 1 *cm*; grain properties are listed in Supplement 3. Existence of MB distributions provides strong evidence that local, peculiar grain dynamics: (i) are nominally Hamiltonian, (ii) determine a canonical equilibrium distribution of *macroscale*, athermal, mechanical energy states, and (iii) can be modeled energetically as a macroscale analog of typical classical atomic-scale systems, i.e., as a superposition of (configuration-independent) individual grain kinetic energies, plus a configuration-dependent (but momentum-independent) elastic-contact potential energy. The grains shown (manufactured by Rosler) are: (**a**) RS19K; (**b**) mixed media; (**c**) RS1010; (**d**) RCP0909; and (**e**) RS3515.

Four pieces of evidence support the proposed equilibrium model: (i) A scaling argument (Supplement 1), focused on the peculiar dynamics of high-restitution grains, demonstrates that on short, near-collision time-scales, N-grain systems remain nominally Hamiltonian, i.e., non-dissipative. (ii) Normalized histograms of two-dimensional peculiar velocity magnitudes, shown in Fig. 2 and measured at the grain pile surface, are well-fit by Maxwell-Boltzmann probability densities. This result, observed over a wide range of grain shapes, sizes, and densities, strongly indicates existence of local equilibrium within a canonical ensemble. These results are also consistent with the proposed picture of strictly momentum-dependent kinetic energies and (nominally) momentum-independent grain collisional/elastic potential energies; existence of the Maxwell-Boltzmann distribution requires both. (iii) A rough test of the equipartition theorem, applied to the measured mean random grain kinetic energy, *m*_*g*_〈**v**′ · **v**′〉/2, shows that the average kinetic energy per grain remains roughly fixed for all eight of the grain types tested, a result expected for systems of grains having similar mass, size, shape, and elastic properties, and subjected to a fixed vibrational energy input. Equipartition of grain kinetic energy is likewise indicated by the results in Fig. 2, which show approximately equal variances, 〈**v**′ · **v**′〉, for all grains tested. Importantly, equipartition: (a) implies Hamiltonian dynamics, (b) indicates weak influence of grain momentum on system potential energy, and (c) applies even though our grains exist in high-density gas and liquid states (see below). (iv) On time-scales long relative to the grain collision time-scale, individual grains exhibit memory-free Langevin dynamics (see below) which means that high-restitution grains subject to low-amplitude vibration, at all times and all locations, always tend toward local equilibrium states.

### Short-Time, Single-Grain Fluctuations: Particle-in-a-Cage Effect

**Figure 3:**
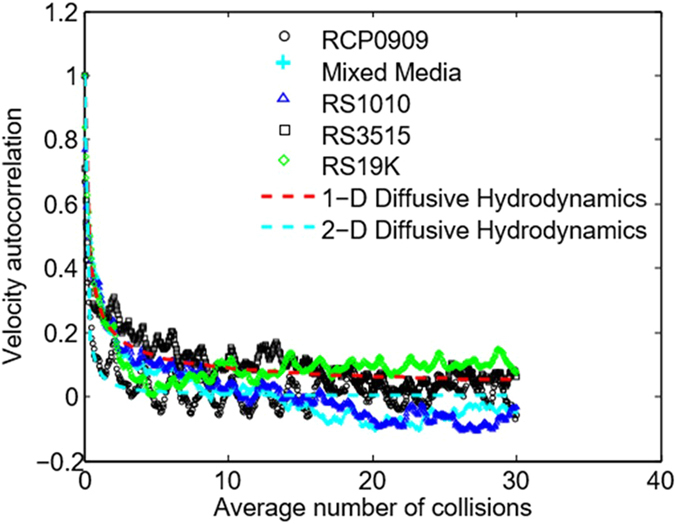
Solid-liquid, liquid, and dense gas single-grain hydrodynamics. The velocity autocorrelation function, *ψ* = *ψ*(*N*_*c*_), for single *virtual* grains is plotted as a function of the characteristic number of grain collisions, 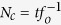, where *t* is the time lag. PIV velocity measurements are obtained at the interrogation area shown in Fig. 2f; *ψ*′*s* are those associated with the corresponding filtered, peculiar, local grain velocity. Filtering was used to remove the velocity contribution, i.e., the spectral peaks, produced by the solid-like vibrational response of the grain pile-bowl system. A non-solid-like subharmonic mode at 14.6 Hz - see text and Fig. 5 - was not filtered. Although approximately 10 to 20 grains pass through the interrogation window during any given ≈10 second experiment, on time-scales longer than 10^−3^ s, each grain behaves as a memory-free Brownian particle, immersed in a discontinuous bath of small, high-frequency elastic bumps; see text and Supplement 2. Thus, measuring the velocity of a set of grains passing through a fixed interrogation window, using a sample frequency, *f*_*s*_, of 500 Hz^23^, is equivalent to (virtually) tracking and measuring the velocity of a single grain (at the same *f*_*s*_). Physically, we can roughly differentiate three thermodynamic grain states: (i) a dense gas state corresponding to grains whose *ψ*′*s* remain always positive (RS3515 and RS19K); (ii) a liquid state for which *ψ* dips below, but then subsequently approaches zero (RCP0909); and (iii) a mixed solid-liquid state for which *ψ* drops below, but doesn’t reapproach zero (RS1010, mixed media). The influence of hydrodynamic organization of short-time-scale single-grain dynamics, which takes place over 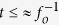 - see Fig. 4 - decays rapidly on the long time-scales shown here.

**Figure 4:**
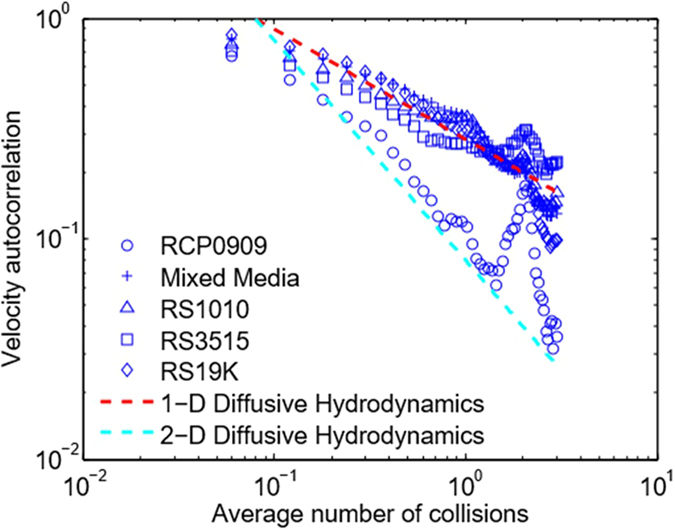
Hydrodynamic organization of short-time-scale single-grain dynamics. High-frequency resonant elastic modes are collisionally excited within all grains in any vibrated grain pile. On time-scales long relative to the inverse fundamental frequency, 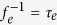, continuum conservation equations governing the ensemble (or equivalently, time) average grain *flow* can be rigorously derived; see Supplement 2. As argued in Supplement 2, the local hydrodynamic (continuum) response of the grain fluid to vibration-driven injection of momentum produces single-grain *ψ*′*s* – on time-scales long relative to *τ*_*e*_, but not exceeding the characteristic collision time, 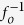 – that decay as *t*^−*d*/2^, where *d* is the effective dimensionality of single-grain peculiar dynamics. PIV measurements show that *d* ≈ 1 for seven of the eight grains tested, but that *d* ≈ 2 for RCP0909. Lines having slopes of −1 (red) and −1/2 (blue) have been inserted.

In contrast to scattering studies of molecular hydrodynamic systems, PIV measurements of vibrated grain dynamics allow nearly-direct measurement of single-particle dynamics, as embodied in the velocity autocorrelation function, 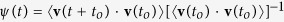, where **v**(*t*) is the instantaneous peculiar particle velocity. Importantly, we observe two well-known signatures of sub-collision and supra-collision single-atom dynamics in our vibrated grain system: (i) On sub- and near-collision time-scales, for four of the eight grains tested, we observe direct evidence of the ‘particle-in-a-cage’ effect predicted by molecular dynamics simulations in simple liquids^25^; see Fig. 3. (ii) On time-scales on the order of 0.1*τ*_*c*_ to 3*τ*_*c*_, and for all eight of the grain types tested, we observe algebraic decay in *ψ*(*t*), where, as predicted by molecular dynamics simulations, *ψ*(*t*) decays as *t*^−*d*/2^, and where *d* is the nominal dimension of the random dynamics; see the next section and Fig. 4.

**Figure 5:**
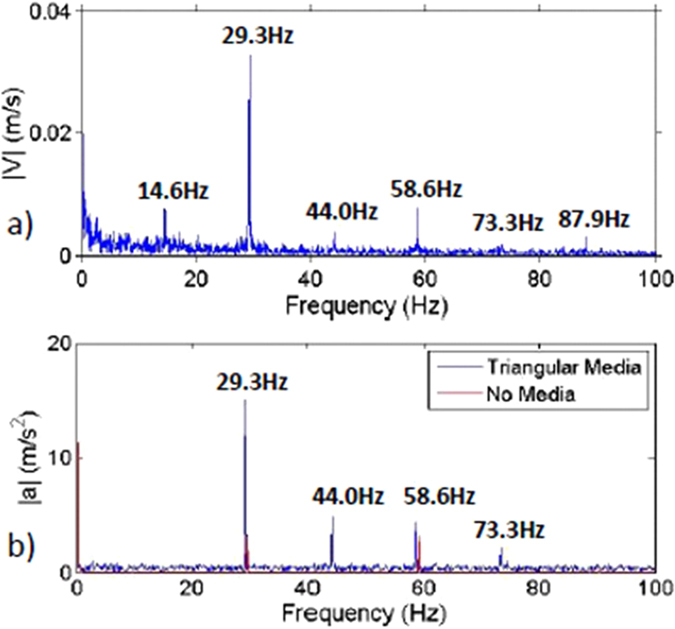
Hydrodynamic grain pile response to vibration. Amplitude spectra^23^ determined from local PIV grain velocity measurements (measurement location shown in Fig. 2f) and from bowl acceleration measurements (taken from bowl exterior) are shown in (**a**,**b**) respectively. Nominally coincident peaks in (**a**,**b**) correspond to resonant acoustic compression waves excited within the solid-like grain pile-bowl system. The fluid-like dynamics of individual grains and of the entire grain pile are studied by filtering out the solid-like acoustic response.

Physically, the trapped-particle effect reflects a collision-time-scale resonant response to injection of momentum, here transmitted throughout the pile by damped hydrodynamic acoustic modes; see below and Fig. 5. At all effective grain densities, short, single-grain-scale, acoustic waves are kicked off from long wavelength longitudinal (compression) modes by collisional momentum transfer. At high number densities, grains remain closely packed so that these short wave modes survive randomization, at least for a time. This effect, signaled by a negative dip in the near-collision time-scale *ψ*(*t*) and representing an approximate indicator of the liquid state, is seen in four of the eight grains tested; see Fig. 3. While predicted by MD simulations, this effect has not been directly observed in atomic liquids.

### Hydrodynamic Organization of Short-Time-Scale Single-Grain Dynamics: t^−d/2^ Decay

On time-scales on the order of 10*τ*_*c*_ to 10^3^*τ*_*c*_, a weak, collective response to the motion of a single molecule is predicted in MD simulations of dense gases^4^,^26^, an effect observed in the dynamics of vibrated grains^11^. Alder and Wainright^4^ showed that the response reflects formation of a molecular-scale vortical wake which induces a weak hydrodynamic flow in the direction of particle motion. Significantly, long-time correlated motion of individual atoms, indicated by slow decay of *ψ*(*t*), contradicts a central premise of classical transport theory^3^ in which rapid collisional randomization destroys all collective, hydrodynamic effects.

Long-time-scale, collective grain hydrodynamics plays a similar role in organizing single-grain dynamics, with three noteworthy differences. First, as shown in Fig. 4, the organizing action occurs over sub- and near-collision time-scales, 0.1 <~ t* <~ 3, where t* = *t*/*τ*_*c*_. In dense gas systems, modeled as hard spheres^3^,^4^, algebraic *t*^−*d*/2^ decay occurs over 4 <~ t* <~ 12. Second, observed *t*^−1/2^ and *t*^−1^ decays in Fig. 4 appear to reflect local, diffusive transport of momentum in response to cyclic vibrational forcing. As shown in Supplement 2, on time-scales on the order of *τ*_*c*_, the hydrodynamic response of the granular fluid to a single injection of momentum (at the end of any given vibration cycle) produces a grain autocorrelation function that decays as *t*^−1/2^ for grains experiencing predominantly one-dimensional random motion, and as *t*^−1^ for grains undergoing two-dimensional random motion. Velocity measurements show that seven of the eight grains tested fall into the first category, while one, high-density RCP0909, falls into the latter. The results in Fig. 4 are thus consistent with short-time-scale hydrodynamic organization of grain-scale dynamics through diffusion of injected momentum. Third, algebraic short- (0.1 <~ t* <~ 1) and intermediate- (1 <~ t* <~ 3) time-scale decay occurs at effective densities where the grain fluid behaves not only as a high-density gas -see the plots for *ψ*(*t*) in Fig. 3 for ‘RS19K’ and ‘RS3515’ - but also as a liquid and two-phase liquid-solid mixture (indicated, respectively, by negative autocorrelations that reapproach and do not reapproach zero) - see the plots for ‘RCP0909’, ‘mixed media’, and ‘RS1010’ in Fig. 3. The latter observation raises the question of whether hydrodynamic organization of short- and intermediate-time-scale single-atom dynamics also occurs in atomic liquids. While simulations have apparently not exposed this effect, due to the highly interconnected nature of the atomic-scale liquid state - difficult to capture in simulations - it is not implausible that short-time hydrodynamic organization *does* occur.

As a closing aside, we note that the weak, near-cyclic variations in the autocorrelations shown in Fig. 3 arise due to the presence of an unfiltered, fluid-like, subharmonic inertial response to vibrational forcing; see next subsection. This feature manifests itself on short, near-collision-time-scales as an up-and-down variation in the autocorrelation, making its first appearance at essentially the same time lag (approximately 1 to 3 collision times) for all grains tested; see Fig. 4.

### Hydrodynamic Modes

**Figure 6:**
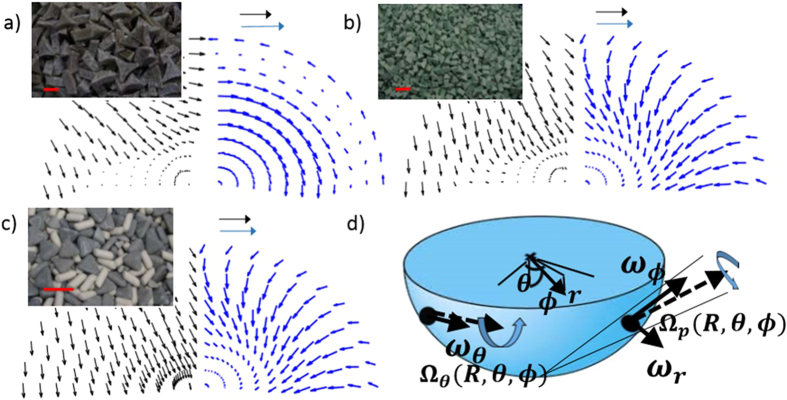
Qualitative comparison of measured and calculated hydrodynamic grain flows. Measured, time-averaged surface flow fields are shown in black. Theoretical flow fields, calculated using the hydrodynamic conservation equations derived in Supplement 2 (and corresponding, in the present model, to the Navier-Stokes equations), are shown in blue. The simplified model solves the low Reynolds number vorticity equation within a hemisphere subjected to the same multi-modal vibrational forcing used in our experiments. Since actual and model bowl surfaces are smooth, the granular fluid slips rather than sticks at the bowl boundary. Thus, a new vorticity boundary condition, designed to capture the time-averaged wall-to-grain transfer of rotational momentum, must be introduced; see Supplement 2. Experimentally measured effective grain kinematic viscosities (Supplement 2), *ν*_*e*_, and characteristic experimental and calculated velocity scales, *V*_*e*_ and *V*_*m*_, shown as horizontal black and blue arrows, respectively, are as follows: (**a**) RS3515DZS, *ν*_*e*_ = 0.021 m^2^ s^−1^, *V*_*e*_ = *V*_*m*_ = 6.3(10^−4^) ms^−1^; (**b**) RS1010, *ν*_*e*_ = 0.0049 m^2^ s^−1^; *V*_*e*_ = *V*_*m*_ = 3.2(10^−3^) ms^−1^; (**c**) mixed media, *ν*_*e*_ = 0.0050 m^2^ s^−1^; *V*_*e*_ = *V*_*m*_ = 3.2(10^−3^) ms^−1^; (**d**) Position-dependent (time-average) wall vorticity, (*ω*_*r*_, *ω*_*ϕ*_, *ω*_*θ*_), produced by bowl rotation vectors, Ω_*p*_, and Ω_*θ*_. Red scales equal 1 cm.

In response to small perturbations, vibrated grain piles exhibit multi- modal hydrodynamic responses similar to those observed and predicted in atomic liquids^3^,^6^,^22^. As an essential preliminary, Supplement 2 outlines a course-graining procedure, a macroscale adaptation of a microscale argument^6^, for recasting mass, momentum, and energy conservation laws, applied to systems of discrete individual grains, into long-time-scale continuum form. Derivation of the hydrodynamic conservation equations represents an important step, allowing not only interpretation of the grain pile’s hydrodynamic modal response to vibration, but more generally, modeling of long-time-average, vibration-driven grain flow; see below and Fig. 6.

Amplitude spectra determined from simultaneous bowl acceleration and PIV grain velocity measurements, shown in Fig. 5, expose several hydrodynamic features either experimentally observed or simulated, or expected in atomic liquids and dense gases: (i) damped acoustic modes, (ii) long-time-average fluid flow, and (iii) generalized Langevin single-grain dynamics. To guide the analysis, we express the instantaneous azimuthal or radial velocity, *v*_*i*_(**r**, *t*), *i* = 1, 2, of any given grain as a superposition of the velocity contribution produced by in-pile acoustic modes, 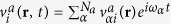, the local long-time-average bulk flow velocity, 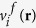, obtained by filtering the acoustic modes from the measured velocity at point **r**, followed by time averaging, and the random peculiar velocity produced by inter-granular collisions, 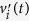. Thus, 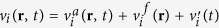. In addition, based on experimental observations above, and consistent with single-atom peculiar dynamics^3^,^7^, we assume individual grain dynamics can be modeled by the generalized Langevin equation: 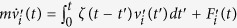, where *ζ*(*t* − *t*′) is a history-dependent friction factor, taken as isotropic, and 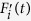 is the random collisional force.

Considering first in-pile acoustic modes, Fig. 5b shows that the vibrated grain pile and bowl behave as a coherent, solid-like body: while the empty bowl vibrates at the driving frequency of *f*_*o*_ = 29.3 Hz, as well as the second harmonic (2*f*_*o*_ = 58.6 Hz), the bowl and grain system vibrate at the first three bowl harmonics, as well as a subharmonic of *f*_1/2_ = 14.6 Hz. In addition, combinations of *f*_*o*_ and *f*_1/2_ are apparent. [The subharmonic appears to be an inertial effect, where the grain pile responds to a vibrational kick in the azimuthal direction of the bowl, injected over a portion of the azimuthal vibration cycle, but then remains suspended or in continued azimuthal motion over the remainder of the cycle]. In terms of the assumed grain velocity decomposition, observed spectral peaks in Fig. 5 correspond to delta functions in the Fourier transform of the acoustic contribution, 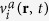. Likewise, the long-time-average velocity, 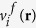, measured at the interrogation point, **r**, makes another delta function contribution at *ω* = 0.

A simple scaling argument, using a vibrated annular cylinder as a proxy, indicates that observed acoustic modes (excluding the subharmonic at 14.6 Hz) correspond to resonant standing waves excited across the half-toroid-like bowl, where the wavelength of each succeeding higher-order harmonic is halved. Based on this picture, the effective in-pile sound speed is, as required, fixed, yet surprisingly small, only on the order of 10 m/s. Thus, observed acoustic modes, which are viscously damped given the rapid relaxation to rest following cessation of vibration, correspond to viscously damped acoustic modes observed in scattering experiments in atomic fluids^3^,^6^. (Equally significant, the approximate two-order-of-magnitude difference between the effective in-pile sound speed and local bulk velocity ensures maintenance of local macroscale equilibrium, conforming to similar dynamics in atomic liquids).

Considering finally the random motion of individual grains, as represented by 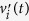, the near-zero-frequency spectrum in Fig. 5, combined with a scaling argument, reveals two significant features. First, individual grains, on hydrodynamic time-scales, 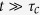, are subject to memory-free Langevin dynamics. This is shown as follows: (i) Similar to individual atoms in dense atomic fluids^3^, we note that on sub- and near-collision time-scales, individual grains are subject to generalized Langevin (GLE, memory-dependent) dynamics. This is clearly indicated by the short time-scale dynamics shown in Fig. 4, which can only be explained via a GLE model (details not shown). (ii) Taking the Laplace transform of the GLE and focusing on the hydrodynamic limit^3^,^6^, we obtain a near-zero-frequency Lorentzian. Second, a scaling argument shows that high-frequency, solid-phase elastic vibration modes are collisionally excited within individual grains; these, in turn, produce small, high-frequency elastic bumps on each grain surface. On time-scales on the order of the collision frequency and longer, the collection of these high-frequency surface perturbations functions as a discontinuous sea of bath particles, producing Brownian grain dynamics. Since the characteristic frequencies of these internal elastic modes are approximately an order of magnitude higher than *f*_*o*_, we idealize the random force, 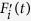, above as delta correlated. Thus, consistent with Fig. 5a, the combined signatures of LE dynamics and small-magnitude, delta-correlated elastic contact forces produces a small-amplitude white noise spectrum, superposed on a near-zero-frequency Lorentzian.

### Time-Averaged Grain Flow

Vibrated grain piles exhibit the same dynamical hierarchy observed in dense gases and simple liquids: (i) on sub-, near-, and supra-collision-time-scales, generalized Langevin single-grain response and low-frequency, multiple-grain, collective hydrodynamic response, here to vibrational forcing, and (ii) as described in this section, on longer time-scales, organized, emergent flow.

We solve the long-time-scale Navier-Stokes equations, subject to the time-averaged rotational forcing used in our experiments, and compare the calculated time-average flow against our observations, with typical results shown in Fig. 6. In order to simplify the calculation, we model the half-toroidal bowl as a hemisphere. Simple scaling shows that due to the small inner radius of the bowl, associated time-averaged vorticity generation, determined by the rotational velocity of the bowl surface, is much weaker on the inner bowl radius than on the outer. Using the approximate, analytical model outlined in Supplement 2, we obtain three-dimensional, helical flows that are qualitatively similar to those measured.

## Summary

Experiments and statistical mechanical modeling demonstrate that high-restitution grain piles, subject to low-amplitude vibration, share many essential dynamical properties known and predicted in molecular hydrodynamic dense gas and liquid systems: (i) Grain systems exist in local (macroscale) statistical mechanical equilibrium. (ii) On sub- and near-collision-time-scales, single grains exhibit strong, hydrodynamically-induced organization, analogous to the ‘long-time-tail’ effect predicted in dense gases. (iii) On the same time-scales, individual grains undergo generalized Langevin dynamics. (iv) On longer time-scales, 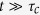, individual grains, bathed in a discontinuous bath of high-frequency, in-grain elastic contact points, experience memory-free Langevin dynamics. (v) On the same long time-scales, the linear hydrodynamic response of grain piles to vibrational forcing takes place through damped acoustic modes. Although not discussed, analysis also suggests that a purely diffusive, but weak entropy mode, as well as strong vorticity modes, as observed and predicted in molecular fluids^3^,^6^, are also excited. (vi) Finally, on long time-scales 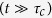, well-ordered granular flow fields set up. A simplified model which solves the Navier-Stokes equations, provides qualitatively consistent predictions of observed grain flows.

## Additional Information

**How to cite this article**: Keanini, R. G. *et al*. Macroscopic liquid-state molecular hydrodynamics. *Sci. Rep.* **7**, 41658; doi: 10.1038/srep41658 (2017).

**Publisher's note:** Springer Nature remains neutral with regard to jurisdictional claims in published maps and institutional affiliations.

## Supplementary Material

Supplementary Information

## References

[b1] Berne B. J. & Pecora R. Dynamic light scattering (Wiley, 1976).

[b2] Lovesey S. W. In Dynamics of solids and liquids by neutron scattering, Lovesey S. W. & Springer T. eds (Springer-Verlag, 1977).

[b3] Boon J. P. & Yip S. Molecular hydrodynamics (McGraw-Hill, 1980).

[b4] Wainwright T. E., Alder B. J. & Gass D. M. Decay of time correlations in two dimensions. Phys. Rev. A A4, 233–236 (1971).

[b5] Berne B. J. & Forster D. Topics in time-dependent statistical mechanics. Ann. Rev. Phys. Chem. 22, 563–596 (1971).

[b6] Forster D. Hydrodynamic fluctuations, broken symmetry, and correlation functions (Perseus, 1990).

[b7] Evans D. J. & Morriss G. P. Statistical mechanics of nonequilibrium liquids (ANU E Press, 2007).

[b8] Brown J. C., Pusey P. N., Goodwin J. W. & Ottewill R. H. Light scattering study of dynamic and time-averaged correlations in dispersions of charged particles. J. Phys. A: Math Gen. 8, 664–682 (1975).

[b9] Pusey P. N. Intensity fluctuation spectroscopy of charged Brownian particles: the coherent scattering function. J. Phys. A: Math. Gen. 11, 119–136 (1978).

[b10] Shattuck M. D., Ingale R. A. & Reis P. M. Granular thermodynamics. Powders and Grains 2009, Proc. 6th Int. Conf. Micromechanisms Granular Media, Nakagawa M. & Luding S. eds, Amer. Inst. Phys. 43–50 (2009).

[b11] Reis P. M., Ingale R. A. & Shattuck M. D. Caging effects in a granular fluid. Phys. Rev. Lett. 98, 188301 (2007). 17501613 10.1103/PhysRevLett.98.188301

[b12] Marty G. & Dauchot O. Subdiffusion and cage effect in a sheared granular material. Phys. Rev. Lett. 94, 015701 (2005). 15698097 10.1103/PhysRevLett.94.015701

[b13] Campbell C. S. Granular material flows - an overview. Powder Tech. 162, 208–229 (2006).

[b14] Gibbs J. W. Elementary principles in statistical mechanics (University Press, 1902).

[b15] Landau L. D. & Lifshitz E. M. Statistical physics vol. 5, 3rd ed. (Oxford 1969).

[b16] Pathria R. K. & Beale P. D. Statistical mechanics, 3rd ed. (Elsevier, 2011).

[b17] Jenkins J. T. & Savage S. T. A theory for the rapid flow of identical, smooth, nearly elastic, spherical particles. J. Fluid Mech. 130, 187–202 (1983).

[b18] Zwanzig R. Time-correlation functions and transport coefficients in statistical mechanics. Ann. Rev. Phys. Chem. 16, 67–102 (1965).

[b19] Kadanoff L. P. & Martin P. C. Hydrodynamic equations and correlation functions. Annals Phys. 24, 419–469 (1963).

[b20] Kubo R. Statistical-mechanical theory of irreversible processes. I. J. Phys. Soc. Japan 12, 570–586 (1957).

[b21] Kubo R., Toda M. & Hashitsume N. Statistical physics II: nonequilibrium statistical mechanics (Springer, 1991).

[b22] Mountain R. D. Generalized hydrodynamics. Advances in Molecular Relaxation Processes 9, 225–291 (1976).

[b23] Fleischhauer E., Azimi F., Tkacik P., Keanini R. & Mullany B. Application of particle imaging velocimetry (PIV) to vibrational finishing. J. Materials Proc. Tech. 229, 322–328 (2016).

[b24] Toda M., Kubo R. & Saito N. Statistical physics I, 2nd ed. (Springer-Verlag, 1992).

[b25] Levesque D. & Verlet L. Computer “experiments” on classical fluids, III. time-dependent self correlation functions. Phys. Rev. A 2, 2514–2528 (1970).

[b26] Levesque D. & Ashurst W. T. Long-time behavior of the velocity autocorrelation function for a fluid of soft repulsive particles. Phys. Rev. Lett. 33, 277–280 (1970).

