# Supplementary material for: Macroscopic liquid-state molecular hydrodynamics
Source: Sci Rep. 2017 Jan 31;7:41658. doi: 10.1038/srep41658 (PMC5282555; doi:10.1038/srep41658)
Supplement: Supplementary Information [file srep41658-s1.pdf]

Macroscopic Liquid-State Molecular Hydrodynamics

Supplements

R. G. Keanini, Peter T. Tkacik, Eric Fleischauer,
Hossein Shahinian, Jodie Sholar, Farzad Azimi and Brid Mullany

Department of Mechanical Engineeering and Engineering Science
The University of North Carolina at Charlotte
Charlotte, North Carolina 28223-0001

Supplement 1: Equilibrium statistical mechanics and thermodynamics models

R. G. Keanini, Peter T. Tkacik, Eric Fleischauer,

Hossein Shahinian, Jodie Sholar, Farzad Azimi and Brid Mullany

Department of Mechanical Engineering and Engineering Science

The University of North Carolina at Charlotte

Charlotte, North Carolina 28223-0001

Macroscale equilibrium statistical mechanics model

The equilibrium model applies to the peculiar dynamics of a system of N high restitution grains undergoing low amplitude vibration. Experimentally, the random/peculiar component of grain motion is obtained by filtering the acoustic hydrodynamic modes shown in the velocity spectrum, Fig. 5, and subsequently removing the (local) long-time-average bulk grain velocity, $\langle \mathbf{v}(\mathbf{r}) \rangle = (N_s \Delta t_s)^{-1} \sum_j \mathbf{v}(\mathbf{r}, t_j)$, where N_s and Δt_s are, respectively, the number of velocity measurements obtained and the sample interval, and where $\mathbf{r}$ corresponds to the centroid of the interrogation area used in PIV-based velocity measurements; see Fig. 2f.

The equilibrium statistical mechanics model rests on five assumptions, the first, third, and fourth of which are standard in microscale equilibrium models: i) Over sub-collision to supra-

collision-time scales, e.g., $0 < t \sim 3\ \tau_c - 5\ \tau_c$, where $\tau_c = f_o^{-1}$ and f_o is the vibration forcing frequency, grain dynamics are Hamiltonian. A scaling argument below shows that for low-amplitude vibration of high-restitution grains, this is a reasonable simplification. ii) For any given family of grains characterized by an equivalent grain diameter, $d_{g,e} = \left(6\pi^{-1}\text{volume}\right)^{-1/3}$, and for any grain shape and any grain mass density within this family, we assume that a macroscale, length-scale-dependent Boltzmann constant, $k_e = k_e\left(l_e\right)$, exists. Since k_e represents a conversion factor between temperature and energy, the assumption of a constant macroscale k_e is equivalent to defining a temperature scale.[1] iii) Upon any given observation, the N-grain system will be found, with equal probability, in any of the athermal, purely mechanical Ω *grain-scale energy states* available to it. iv) At equilibrium, the observed, *thermodynamic state* of the N-grain system corresponds to that state having the maximum number of associated grain-scale energy states. v) The N-grain system remains in a single, fluid-like state, i.e., local, fixed or transient solidification zones do not appear. While solidification can be ubiquitous in many grain flow problems, in our experiments, for all eight grain types tested, solidification, at least on the observable free surface, does not occur. A scaling argument below estimates the critical grain depth, for any given set of vibrational conditions and grain material properties, below which grain kinetic energy is insufficient to overcome grain solidification. Based on this rough calculation, solidification does not occur in our system.

Given these assumptions, we first follow standard microscale arguments[2,3] to obtain: i) the macroscale canonical distribution and partition functions, $P\left(E_j\right)$ and $Q\left(V,T,N\right)$, and ii) the macroscale bridge relation, $A = -k_e T ln Q$, where A is the Helmholtz free energy. For clarity, we label the effective macroscale temperature and pressure as T and P, respectively, noting their

definitions below. Thus, assume a canonical ensemble of replica N-grain systems, each exchanging energy with a large, encompassing reservoir. Given the third and fourth assumptions above, a standard argument[2,3] can be used to minimize the function, $W = W(\{n\})$ of distribution sets, $\{n\} = \{n_1, n_2, ..._M\}$, subject to the constraints of fixed total (ensemble) energy, $\tilde{E} = \sum_{i=1}^{M} n_i E_i$, and fixed total grain number, $\tilde{N} = \sum_{i=1}^{M} n_i$. Here, n_j is the number of replica systems observed (at any instant) in macroscale energy state E_j, and $M = \tilde{E}/E_1$ corresponds to the observation in which all $\tilde{N}$ replicas are found in the lowest accessible energy state, E_1. The resulting equilibrium distribution, $p_i = p(n_i) = \exp(-\beta_e E_i) Q^{-1}$, $Q = \sum_j^M \exp(\beta_e E_j)$, incorporates a Lagrange multiplier, β_e, which can, in principle, be determined by satisfying the ensemble energy constraint.

Equilibrium thermodynamic model for vibrated grain systems

As in N-particle microscale problems, the N-grain-system equilibrium thermodynamic model requires that the effective system entropy, S, be defined in terms of the number of (athermal, mechanical) grain energy states, Ω, that are accessible to the system, specifically, the number of states within some (uncertainty-determined) interval, $(E - \Delta E, E + \Delta E) : S = k_e ln\Omega$, where again k_e, the effective Boltzmann constant, is determined by how the effective grain temperature scale is defined. Importantly, as in microscale thermodynamics, this definition of S incorporates all of the essential properties of the traditional entropy, at least in fixed mass, fixed- composition/non-reacting systems, apropos confined, high-restitution grain systems undergoing low-amplitude vibration: i) S is maximized at equilibrium, ii) the magnitude of S depends, due to $\Omega's$ dependencies, only on N, the system (time-average) volume, V, and E, iii) due to the statistical independence of subsystems,

subsystem entropies are additive, and iv) S is zero in non-vibrated systems.

Given the microcanonical definition relating S and Ω, or more generally, the canonical relation, $S = -k_e \sum_i p_i ln p_i$, where p_i is given above, the traditional Gibbs thermodynamic framework can be used to self-consistently calculate any effective equilibrium thermodynamic property. Specifically, assuming that the long- time average peculiar dynamics at any given point are stationary, or more to the point, that local, nominally-Maxwellian equilibrium exists, then given a realistic (Hamiltonian) model of N-grain-subsystem dynamics, an associated partition function, $Q = \sum_j^M \exp(\beta_e E_j)$, can be determined, and in turn, used to compute local equilibrium thermodynamic properties.

Hamiltonian dynamics in high-restitution grain piles

A central assumption, on which rests the model of equilibrium statistical mechanics, is that peculiar grain dynamics are nominally Hamiltonian. This assumption, which is well-met in our experiments, at least for steel, aluminum, ceramic, and similar grain materials, requires that the characteristic grain velocity, $v_o = A\omega$, is less than the critical velocity, v_c, separating nominally elastic and elastic-viscoelastic grain collisions;[4] here, A and ω are the characteristic vibration amplitude and frequency, $\omega = 2\pi f_o$. Under these conditions, collisions are nominally elastic and dissipationless, so that collision time-scale grain dynamics can be described in terms of a Hamiltonian.

In order to consider this crucial question in detail, we note that a Hamiltonian statistical mechanical model of high-restitution grains undergoing low amplitude vibration neglects two sources of dissipation: sliding frictional contact between colliding grains, and thermal dissipation of elasto-acoustic energy within grains. A straightforward scale analysis of the equation of motion of indi-

vidual grains within the vibrating pile, stated in the container-fixed, non- inertial frame:

$$m_g \ddot{z} = -m_g \left(g + A\omega^2 \sin \omega t\right) + F_{fric,z} + F_{elast,z} \tag{1}$$

shows that, for the conditions in our experiments:

i) the inertial force, $F_{inertia} = m_g A\omega^2$, is roughly an order of magnitude larger than grain weight: $A\omega^2 g^{-1} = O\left(10\right);$

ii) the ratio of characteristic elastic to inertial forces, $F_{elast,z}\left(m_g A\omega^2\right)^{-1} = O\left(10^{-4}\right)$, where, for Hertzian contact, $F_{elast,z} \approx E^{1/3}\left(A\omega\right)^{4/3} m_g^{2/3} a_c^2 d_g^{-2}$, with the elastic contact area, $a_c \approx \left(\frac{3F'_{inertia} d_g}{8E}\right)^{1/3}$, and where $F'_{inertia} d_g^{-2} \approx m_g \left(A\omega\right)^2 d_g^{-2}$, is the inertial pressure exerted on the grain; and most importantly,

iii) the ratio of characteristic friction to inertial force, $F_{fric,z}\left(m_g A\omega^2\right)^{-1} = O\left(10^{-2}\right)$, where $F_{fric,z} \approx \mu_k m_g (A\omega)^2 d_g^{-1}$, and where $\mu_k \approx 10^{-1}$ is the coefficient of kinetic friction.

Thus, corresponding ratios involving the characteristic grain kinetic energy, elastic potential energy, and frictional energy dissipation, $m_g\left(A\omega\right)^2$, $F_{elast,z}\Delta z_e$, and $F_{fric,z}A$, respectively, exhibit either identical magnitudes, or in the case of $F_{elast,z}\left(m_g\left(A\omega\right)^2\right)^{-1} = O\left(10^{-7}\right)$.

The above estimates apply to grain dynamics that include, and are dominated by, the solid-like vibration of the grain pile- container system. As shown in Fig. 5, grain velocity spectra indicate a strong separation of energy scales, one associated with the solid-like vibrational motion and the other with the fluid-like motion. Thus, repeating the above estimates, but replacing the solid-phase velocity scale, $A\omega$, with the smaller, experimentally observed hydrodynamic, i.e., long-time-averaged grain flow velocity scale, $v_s = O\left(10^{-2}\text{ m/s}\right)$, we obtain essentially the same

results. Thus, Hamiltonian dynamical models are reasonable, at least in the limit of low amplitude vibration of high restitution grain piles. Aside: For simplicity, grain rotational dynamics have not been considered in either the statistical mechanics model, nor in the grain dynamics model.

Grain pile solidification

Vibrating grain beds solidify, i.e., long-time-scale granular flow ceases, at depths exceeding a critical depth, z_c. This depth can be estimated for a vertically vibrating grain column of constant cross-sectional area by balancing the total characteristic kinetic energy of the unsolidified grain layers above z_c against the total elastic potential energy of the solidified and elastically strained grain layers below z_c :

$$\begin{aligned}
\frac{1}{2}\left(N_{tot}-N\right)m_o\tilde{g} &\approx \left(\frac{9}{d_g E^2}\right)^{1/3}(m_o\tilde{g})^{5/3}\frac{1}{d_g^{8/3}}\left[\sum_{j=0}^{N}\left(L_o-z_j\right)^{5/3}dz\right] \qquad \text{or,}\\
\frac{1}{2}\frac{z_c}{d_g}m_o\tilde{g} &= \left(\frac{9}{d_g E^2}\right)^{1/3}(m_o\tilde{g})^{5/3}\frac{1}{d_g^{8/3}}\int_0^{Nd_g}\left(L_o-z\right)^{5/3}dz\\
&= \left(\frac{9}{d_g E^2}\right)^{1/3}(m_o\tilde{g})^{5/3}\frac{1}{d_g^{8/3}}\left(L_o^{8/3}-z_c^{8/3}\right) \qquad (2)
\end{aligned}$$

where $L_o = N_{tot}d_g$ is the total static height of the grain column, N_{tot} is the total number of grain layers, each of thickness d_g, within the column, N the number of solidified layers below z_c, m_o is the total mass of the grains within any layer, and $\tilde{g} = g + A\omega^2$ is the sum of the gravitational and induced accelerations. For the conditions used in our experiments, it is found that solidification does not occur for the grain bed depths, $L_o = O\left(0.3\text{ m}\right)$, used.

Necessary experimental conditions

In order to use a vibrated grain system as a molecular liquid or dense gas analog, two experimental conditions should be met. First, in order to take advantage of the existing theoretical machinery developed for molecular hydrodynamic systems, couched as it is in terms of the pair correlation function, $g(r)$[5,6] one should ensure that peculiar grain dynamics, over regions that are several times larger than the PIV interrogation area (2D problems) or volume (3D), satisfy nominal translational and rotational invariance. Second, in order to reliably reproduce molecular-scale interactions, it is necessary to design intergranular potentials, $u(r)$, that capture those believed to exist in the molecular liquid of interest. For example, the hard sphere potential has served as a realistic, zeroth order model of intermolecular interactions in simple atomic liquids.[5,6] For these liquids, the elastic collisions that characterize the dynamics of high restitution grain piles under low amplitude vibration are expected to produce realistic, predictive single atom dynamics and collective N-atom hydrodynamics.

References

[1] Landau, L. D. & Lifshitz, E. M. *Statistical Physics, 3rd Ed. Part 1.* Elsevier, Oxford (1980).

[2] Pathria, R. K. & Beale, P. D. *Statistical Mechanics, 3rd Ed.* Elsevier, Oxford (2011).

[3] Toda, M., Kubo, R. & Saito, N. Statistical Physics I, 2nd Ed. Springer-Verlag, Berlin (1992).

[4] Burgeson, J. W. Impact Studies on Pure Metals. M.S. Thesis, Florida State Univ. (1955).

[5] Boon, J. P. & Yip, S. *Molecular Hydrodynamics.* McGraw-Hill, New York (1980).

[6] Forster, D. *Hydrodynamic Fluctuations, Broken Symmetry, and Correlation Functions.* Perseus, New York (1990).

Supplement 2: Hydrodynamics of high-restitution grain piles undergoing low-amplitude vibration

R. G. Keanini, Peter T. Tkacik, Eric Fleischauer,
Hossein Shahinian, Jodie Sholar, Farzad Azimi and Brid Mullany
Department of Mechanical Engineering and Engineering Science
The University of North Carolina at Charlotte
Charlotte, North Carolina 28223-0001

Introduction

At minimum, three requirements must be met if vibrated grain piles are to serve as macroscale analogs for studying atomic-scale processes in simple liquids and dense gasses: i) experimentally based, self-consistent grain-scale statistical mechanical models must be developed, ii) continuum, long-time-scale versions of short-time-scale conservation laws must be derived, and iii) self-consistent predictions of observed grain hydrodynamics must be derivable from i) and ii). The first requirement is treated in the Article and Supplement 1. The second and third are tackled here via consideration of four problems:

1) Time averaged PIV measurements and direct observation show that the grain piles in our vibrated bowl move in fluid-like flow patterns. See Fig. 6. Previous studies on gravity-driven[1] boundary-

driven,[2] and vibration-driven grain flows,[3,4] as well as grain flows induced by rotating containers,[5,6] have assumed that the long-time-scale collective dynamics can be modeled using either the fixed-viscosity Navier-Stokes (NS) equations, or, e.g., pressure- and strain-rate-dependent viscosities in the generalized NS equations.[1] In the dense gas/liquid limit, an open problem centers on rigorously connecting short-time, random dynamics of discrete N-grain systems to the emergent dynamics of long-time-scale, liquid-like grain flow. Here, a coarse-graining procedure, borrowed from molecular hydrodynamics,[7,8,9] is outlined for recasting exact conservation laws, applied on short time scales to systems of N randomly interacting grains, into continuum form. Given these exact continuum conservation laws, the generalized Navier-Stokes equations follow by assuming linear expansions for local, ensemble averaged mass, momentum, and energy currents in terms of local, averaged momentum, number, and energy densities, as well as local averaged velocity, and local (equilibrium) pressure and temperature fields.[7,8,9]

2) Contrary to theoretically predicted sub-collision time scale, single atom dynamics in simple liquids and dense gasses,[9] sub- collision time scale, individual grain dynamics are not ballistic, but overwhelmingly determined by collective, long time scale hydrodynamics. We propose a simple hydrodynamic model to explain the dynamics manifested in Fig. 4.

3) In order to further enforce physical consistency between atomic microscale systems and macroscale analogs, any proposed *macroscale* statistical mechanical model must allow calculation, via the Kubo relations,[8,9,10] of non-equilibrium transport coefficients. In vibration-driven grain flows, the effective kinematic viscosity, ν_e, comprising the key transport property, must be calculable. We present a rough scaling estimate of ν_e and show that the estimate is comparable to experimentally measured

effective viscosities.[11]

4) On hydrodynamic time and length scales, dynamical consistency between atomic liquid/dense gas systems and analog grain systems requires that vibration-induced grain flows correspond to those produced in identically vibrated liquids/dense gasses. As described in the Article and as detailed below, hydrodynamic-scale consistency is tested in preliminary fashion by solving the NS equations for vibration-driven flow of a constant viscosity Newtonian fluid in a vibrated hemisphere. A non- trivial problem that we also treat concerns development of continuum boundary conditions appropriate to smooth, vibrated grain container walls.

Hydrodynamic conservation equations

We adapt the molecular-scale arguments in[7,8,12] to derive the instantaneous, grain-scale equations of mass, momentum, and energy conservation. Thus, consider a large volume of elastic grains undergoing low-amplitude, single frequency vibration within a smooth-walled, rigid container. In a reference frame attached to the container, and over time scales that are long relative to the characteristic inter-granular collision rate, $\tau = f_o^{-1}$, where f_o is the vibration frequency, assume that an equilibrium state exists in which no bulk displacement of the grains occurs. At some instant, a small perturbing force acts on one, or a small number of grains simultaneously, producing mass, momentum, and energy currents in the surrounding grains. Under low-amplitude vibration, the perturbing force is assumed large enough to produce a collective response, on length scales that are large relative to characteristic grain size, d_g, but small enough that long-time-scale advective transport remains negligible. Both assumptions are crucial since they allow physically reasonable

definitions of local, spatially varying densities and currents.

Introduce definitions of the local, instantaneous grain number density, $n(\mathbf{r},t)$, momentum density, $\mathbf{g}(\mathbf{r},t)$, and energy density, $e(\mathbf{r},t)$:

$$n(\mathbf{r},t) = \sum_{\kappa} \delta\left(\mathbf{r} - \mathbf{r}_{\kappa}(t)\right) \tag{1}$$

$$\mathbf{g}(\mathbf{r},t) = \sum_{\kappa} \mathbf{P}_{\kappa}(t)\delta\left(\mathbf{r} - \mathbf{r}_{\kappa}(t)\right) \tag{2}$$

$$e(\mathbf{r},t) = \sum_{\kappa} \left[\frac{\mathbf{P}_{\kappa} \cdot \mathbf{P}_{\kappa}}{2m_g} + \frac{1}{2} \sum_{\lambda \neq \kappa} V\left(|\mathbf{r}_{\kappa} - \mathbf{r}_{\lambda}|\right) \right] \delta\left(\mathbf{r} - \mathbf{r}_{\kappa}(t)\right) \tag{3}$$

where $\mathbf{r}_{\kappa}(t)$ and $\mathbf{P}_{\kappa}(t)$, are the instantaneous position and momentum of grain κ, and where the delta function acts as a counter for grains that are within some spherical radius, $\mathbf{r_o}(t)$, of $\mathbf{r}$:

$$\begin{aligned} \delta\left(\mathbf{r} - \mathbf{r}_{\kappa}(t)\right) &= 1 \qquad |\mathbf{r} - \mathbf{r}_{\kappa}| \leq |\mathbf{r_o}(t)| \\ &= 0 \qquad \text{otherwise} \end{aligned} \tag{4}$$

Due to the large size of the grains, N' cannot be arbitrarily large. Rather, constraints must be met when defining N', as well as the time interval, Δt, over which we recast system dynamics into continuum form: i) The characteristic size of the system, $L' \approx {N'}^{1/3} d_g$, must be small relative to characteristic size, L_c, of the container holding the grains. ii) Δt must be long enough to ensure local equilibrium, i.e., $\Delta t >> \tau_c = f_o^{-1}$, but short enough that thermal dispersion (produced by grain peculiar kinetic energy) of the N'-grain system remains negligible. The latter constraint ensures validity of momentum conservation, as applied to the system at any instant, t, and can

be understood by a *physical/intuitive* derivation of the Navier-Stokes equations. [A balance of instantaneous surface and body forces acting on an arbitrary fluid particle, combined with introduction of an assumed linear/Newtonian constitutive relationship between surface stresses and local velocity gradients, leads directly to these equations. Importantly, the derivation fails if the time step used to determine the particle's momentum change is so large that significant thermal particle dispersion occurs.] The time scale for thermal dispersion of the N' grain fluid particle is on the order of $\tau_d = N'^{2/3} d_{g,e}^2 D^{-1}$, where D, the effective self-diffusion coefficient for grains, is connected through the Einstein and Stokes relations (see, e.g.,[13]), $D = k_e T_e \left(6\pi d_{g,e} \mu_e\right)^{-1}$, to the effective grain diameter, $d_{g,e}$, (see below), and the effective grain viscosity, μ_e (see below). Note that $k_e T_e = k_e T_e\left(\mathbf{x}, t\right) = m_g \left\langle v_i'\left(\mathbf{x}, t\right) v_i'\left(\mathbf{x}, t\right)\right\rangle$, where, for example, in strongly two-dimensional, locally isotropic grain flows (like those near the grain pile surface in our experimental system), $i = 1, 2$. [Aside: For modeling purposes, it is often convenient to replace non-spherical grains with equivalent spherical grains. A geometric conversion, expected to provide only semi-quantitative predictions of grain dynamics, follows by, e.g., setting the actual grain volume equal to an equivalent sphere. This then leads to an effective $d_{g,e}$.]

Conservation equations, governing the instantaneous continuum dynamics of an arbitrary system of N' grains lying within $\tilde{\mathbf{r}}$ of $\mathbf{r}$, are derived in wavenumber space, following.[12] This approach assumes a small disturbance to the equilibrium state of an N'-grain system, lying within a large equilibrium reservoir, and explicitly captures the long wavelength, slow time-scale (hydrodynamic) system response. An alternative approach, illustrated here via the mass conservation equation: i) bypasses Fourier transforms, ii) explicitly demonstrates mass, momentum, and energy conservation,

and iii) shows that each term in each continuum conservation law represents a volumetric average over the instantaneous grain system volume, $V_o\left(t\right)$. It is important to note that in both approaches, the smeared delta function, $\delta\left(\mathbf{r}-\mathbf{r}_\kappa(t)\right)$, is the mathematical device that spatially homogenizes discrete number, momentum, and energy densities, allowing recasting of the conservation laws in continuum form.

Thus, consider a system of N' grains lying within a deforming, translating volume $V_o(t)$, having characteristic dimension $\mathbf{r_o}(t) = O\left(N'^{1/3}d_g\right)$, where $V_o(t)$ translates with the local mean velocity, $\mathbf{u}\left(\mathbf{r},t\right) = \langle\sum_\kappa \mathbf{v}_\kappa\rangle$, and where an equilibrium ensemble is used for averaging. The instantaneous mass of grains lying within $V_o\left(t\right)$ is given by

$$M\left(t\right) = \int_{V_o(t)} \sum_\kappa m_\kappa \delta\left(\mathbf{r}' - \mathbf{r}_\kappa(t)\right) dV' \tag{5}$$

Taking the time derivative inside the integral gives

$$\int_{V_o(t)} \sum_\kappa m_\kappa \mathbf{v}_\kappa\left(t\right) \delta_{,\mathbf{r}^\kappa}\left(\mathbf{r}' - \mathbf{r}^\kappa(t)\right) dV' \tag{6}$$

Next, calculate the net mass flux through the surface $A_o\left(t\right)$ enclosing $V_o\left(t\right)$

$$\oint_{A_o(t)} \mathbf{g}' \cdot \mathbf{n}' dA' = \oint_{A_o(t)} \left[\sum_\kappa m_\kappa \mathbf{v}_\kappa\left(t\right) \delta\left(\mathbf{r}' - \mathbf{r}_\kappa(t)\right)\right] \cdot \mathbf{n}' dA' \tag{7}$$

and use the divergence theorem to obtain:

$$\int_{V_o(t)} \nabla' \cdot \mathbf{g}' dV' = \int_{V_o(t)} \left[\sum_{\kappa} m_\kappa \mathbf{v}_\kappa (t) \, \delta_{,\mathbf{r}'} \left(\mathbf{r}' - \mathbf{r}_\kappa(t) \right) \right] dV' \tag{8}$$

Since $\delta_{,\mathbf{r}_\kappa} = -\delta_{,\mathbf{r}'}$ at all instantaneous grain positions, $\mathbf{r}_\kappa (t)$, where subscripts denote partial derivatives, then

$$\int_{V_o(t)} \rho_{,t} dV' + \int_{V_o(t)} \nabla' \cdot \mathbf{g}' dV' = 0 \tag{9}$$

Three results follow from Eq. (9). First, by the Liebnitz theorem, the two terms on the left are identically equal to

$$\frac{M(t)}{dt} = 0$$

proving that the mass of the $N'-$grain system is conserved. Second, by application of the mean value theorem, we obtain the differential mass conservation law:

$$\frac{\partial \rho}{\partial t} + \nabla \cdot \mathbf{g} = 0 \tag{10}$$

which holds at some location, $\mathbf{r}*$, within $V_o(t)$. Third, it is clear that each term in Eq. (10) can be interpreted as the instantaneous volume-average of that term, over $V_o(t)$.

The hydrodynamic momentum and energy conservation laws are most easily derived by transforming to wavenumber space.[9,12] The resulting equations are given by:[8]

$$\frac{\partial \mathbf{g}}{\partial t} + \nabla \cdot \tau = 0 \tag{11}$$

and

$$\frac{\partial e}{\partial t} + \nabla \cdot \mathbf{j^e} = 0 \tag{12}$$

where τ is the momentum current density, or equivalently, the stress tensor:

$$\begin{aligned}
\tau_{ij}\left(\mathbf{r},t\right) &= \sum_{\kappa} \frac{P_i^{\kappa} P_j^{\kappa}}{m} \delta\left(\mathbf{r} - \mathbf{r}_{\kappa}(t)\right) - \\
&- \frac{1}{2} \sum_{\kappa\lambda} r_j^{\kappa\lambda} \phi_i \left(\mathbf{r}^{\kappa\lambda}\right) \cdot \\
&\cdot \int_0^1 \delta\left(\mathbf{r} - \frac{\mathbf{r}^{\kappa} + \mathbf{r}^{\lambda}}{2} - \frac{l'}{2}\mathbf{r}^{\kappa\lambda}\right) dl'
\end{aligned} \tag{13}$$

and where $\mathbf{j^e}$ is the energy current density:

$$\begin{aligned}
j_i^e\left(\mathbf{r},t\right) &= \sum_{\kappa} \left[\frac{P_i^{\kappa} P_i^{\kappa}}{2m} + \frac{1}{2} \sum_{\lambda\neq\kappa} \phi\left(\mathbf{r}^{\kappa\lambda}\right)\right] \cdot \\
&\cdot \frac{P_i^{\kappa}}{m} \delta\left(\mathbf{r} - \mathbf{r}^{\kappa}(t)\right) - \sum_{\kappa\lambda,\kappa\neq\lambda} \frac{1}{4m} \\
&r_i^{\kappa\lambda} \phi_j \left(\mathbf{r}^{\kappa\lambda}\right) \left(P_j^{\kappa} + P_j^{\lambda}\right) \cdot \\
&\cdot \int_0^1 \delta\left(\mathbf{r} - \frac{\mathbf{r}^{\kappa} + \mathbf{r}^{\lambda}}{2} - \frac{l'}{2}\mathbf{r}^{\kappa\lambda}(t)\right) dl'
\end{aligned} \tag{14}$$

Here, $\phi\left(|\mathbf{r}^{\kappa\lambda}|, t\right) = \phi\left(|\mathbf{r}^{\kappa}(t) - \mathbf{r}^{\lambda}(t)|\right)$ is the elastic potential energy between pairs of directly contacting grains, as well as between pairs of separated grains, instantaneously connected through randomly connecting and disconnecting chains of elastic contact points. In order to take advantage of the machinery developed for pairwise potentials, we express the elastic potential in this fashion. Likewise, $\phi_i\left(\mathbf{r}^{\kappa\lambda}\right)$ is the ith component of the corresponding elastic force between directly contacting and indirectly contacting grain pairs.

Given exact continuum conservation laws, the generalized Navier-Stokes equations follow by expanding long-time/ensemble-averaged (local) mass, momentum, and energy current densities in terms of both non-derivative reactive terms, which follow from Galilean transformation between the local bulk fluid motion and the local rest state, and derivative dissipative terms which capture, e.g., Fourier thermal conduction and Newtonian frictional/viscous stresses. See.[7,8]

Hydrodynamic organization of sub- and near-collision time scale single grain dynamics

Due to random collisions between grains, sustained, high frequency elastic modes are excited in each grain within the pile. The elastic modes, in turn, produce small, high frequency bumps on each grain surface. Scaling shows that the characteristic contact time between pairs of colliding grains, $\tau_c \approx f_o^{-1}$, is much longer than the (slowest) time scale, τ_E, associated with bump formation. Thus, in terms of single grain dynamics, and on the long collision time scale, τ_c, individual grains can be modeled as memory-free Brownian particles immersed in a discontinuous bath of randomly impinging elastic bumps.

This picture suggests the following hydrodynamic model of multiple grain dynamics, applicable on time scales on the order of the collision time scale, τ_c, but long relative to τ_E. Consider a continuous (and virtual) grain fluid composed of differential grain fluid elements. The dynamics of the grain fluid are determined by the hydrodynamic (NS) equations derived above:

$$v'_{i,t} = \nu_e \nabla^2 v'_i + S_i \tag{15}$$

where $v'_i = v'_i(\mathbf{r}, t)$ is the ith component of the local grain fluid velocity relative to the local average fluid velocity and $S_i = S_i(\mathbf{r}, t)$ is a volumetric momentum source. The pressure gradient term in Eq. (15) has been dropped: we are modeling (experimentally accessible) surface and near-surface grain hydrodynamics, where the hydrodynamic pressure is nominally fixed at ambient.

Concerning the source term, S_i, over the course of any given vibration cycle, any given grain remains in contact with a (small) number of neighboring grains; at each contact point, the grain is acted upon by small, high-frequency elastic bumps. Over time scales greater than τ_E, but less than or on the order of τ_c, we thus model the effect of intergranular elastic contact, which acts at all times on all grains within the pile, as an isotropic, δ function momentum source:

$$S_i = S_i\left(\tilde{\mathbf{r}}, \tilde{t}\right) = a_{e,i}\delta\left(\tilde{\mathbf{r}}\right)\delta\left(\tilde{t}\right) \tag{16}$$

where $\tilde{\mathbf{r}} = \mathbf{r} - \mathbf{r}'$, $\tilde{t} = t - t'$, (r', t') is the location and instant of momentum injection, and $a_{e,i}$ is the average, elastic-contact-induced momentum source strength. Given Eq. (16), the time- and space-dependent response of the granular fluid to vibration-driven momentum injection follows from Eq. (15):

$$v'_i\left(\tilde{\mathbf{r}}, \tilde{t}\right) = \frac{a_{e,i}}{\left(4\pi\nu_e\tilde{t}\right)^{d/2}} \exp\left[\frac{-\tilde{\mathbf{r}}\cdot\tilde{\mathbf{r}}}{4\nu_e\tilde{t}}\right] \tag{17}$$

where d, is the dimension of the hydrodynamic (response) flow.

Turning to calculation of the single grain velocity autocorrelation function,

$$\psi\left(\tilde{t}\right) = \left\langle \mathbf{v_g}(0)\cdot\mathbf{v_g}(0)\right\rangle^{-1}\left\langle \mathbf{v_g}\left(\tilde{t}\right)\cdot\mathbf{v_g}(0)\right\rangle \tag{18}$$

and again focusing on short sub- and near-collision time scales, $\tilde{t}$, where $\tau_E << \tilde{t} = O(\tau_c)$, we approximate the ensemble average, $\langle \mathbf{v_g}(\tilde{t}) \cdot \mathbf{v_g}(0) \rangle$, as

$$\langle \mathbf{v_g}(\tilde{t}) \cdot \mathbf{v_g}(0) \rangle \approx \frac{a_e^2}{\left(4\pi\nu_e\tilde{t}\right)^{d/2}} \exp\left[\frac{-\tilde{\mathbf{r}} \cdot \tilde{\mathbf{r}}}{4\nu_e\tilde{t}}\right] \tag{19}$$

where $\tilde{\mathbf{r}} = \mathbf{r} - \tilde{\mathbf{r}}_\mathbf{o}$, and $\tilde{\mathbf{r}}_\mathbf{o}$ is the grain's initial position. In other words, the single-grain ensemble average velocity at time $\tilde{t}$ is approximated as the grain fluid velocity at the same instant, while the initial grain velocity is approximated as the fluid velocity at the instant of vibration-induced momentum injection, $\mathbf{v_g}(0) \approx \mathbf{v}'\left(\tilde{\mathbf{r}_\mathbf{o}}, \tilde{t} = 0\right) = \mathbf{v_e} \approx \mathbf{a_e}\tau_E$.

Finally, due to the short time scale, single grain displacement is small, i.e., $\tilde{\mathbf{r}}_\mathbf{o} \approx \mathbf{0}$, so that

$$\psi(\tilde{t}) \propto \tilde{t}^{-d/2} \qquad\qquad \tau_E << \tilde{t} = O(\tau_c) \tag{20}$$

thus providing a purely hydrodynamic mechanism - pure diffusion of vibration-induced momentum injection - for explaining the short-time, single grain dynamics exposed in Fig. 4.

Consistency check 1: Scaling estimate of the effective grain viscosity via a macroscale Kubo relation

Here, a Kubo relation[10] is used in a scaling estimate of the grain fluid's effective shear viscosity, μ_e, which, in turn, is compared against experimentally measured viscosities.[11] This represents a significant consistency check since the Kubo relations: i) assume existence of hydrodynamic-

scale conservation laws associated with conserved microscale variables,[7,9] ii) assume existence of a Hamiltonian N-particle system, initially in thermodynamic equilibrium, but subject to small, non-equilibrium fluctuations or forcing, iii) rigorously determine hydrodynamic transport coefficients in terms of correlation functions of current densities, iv) require equilibrium ensemble averaging, and v) connect long-time- and long-length-scale particle dynamics, typically beyond direct observation in microscale systems, to directly observable continuum dynamics.

At least three approaches have been used to derive the Kubo relation for μ_e.[8,9,10,12,14,15] Using any of these approaches, the shear viscosity can be expressed as the cumulative, long-time evolution of the grain-scale transverse momentum current, i.e., shear stress, arising in response to a perturbation to the transverse momentum density:[9]

$$\eta = \frac{\beta}{V} \int_0^\infty \langle \tau_{xy}(t)\, \tau_{xy}(0) \rangle \, dt \tag{21}$$

where the transverse grain-scale stress tensor component is given by

$$\tau_{xy}(t) = \sum_\kappa \left[v_x^\kappa(t)\, P_y^\kappa(t) + \frac{1}{2} \sum_\lambda \left(\mathbf{r}^{\kappa\lambda} \right)_x \frac{\partial u^{\kappa\lambda}}{\partial r_y^\alpha} \right] \tag{22}$$

Here, the random or imposed perturbation acts in the $x-$direction, y is a transverse direction, $\mathbf{r}^{\kappa\lambda} = |\mathbf{r}^\lambda - \mathbf{r}^\kappa|$ is the spatial separation between grains κ and λ, $\mathbf{v}^\kappa(t)$ and $\mathbf{P}^\lambda(t)$ are the instantaneous velocity and momentum of grains κ and λ, relative to the local mean velocity, and $u^{\kappa\lambda}$ represents the separation-dependent elastic potential between grain pairs. See above for a detailed version of τ.

In order to apply (21) to a grain pile undergoing low-amplitude vibration, we first use the equipartition theorem to estimate β_e, the Lagrange multiplier determined by satisfying the canonical ensemble energy constraint. Thus, model pair-wise elastic contact between grain pairs via equivalent elastic springs, note that the corresponding N-grain Hamiltonian is quadratic in its 3N momenta and 3N spatial coordinates, and use the equipartition theorem - see, e.g.,[16] for a generic development - to obtain:

$$\langle H \rangle = 3N\beta_e^{-1} \sim NA^2\omega^2 \tag{23}$$

where A and ω are respectively the vibration amplitude and frequency. Thus, as in microscale problems, the macroscale single-grain energy scale, β_e^{-1}, is on the order of the average kinetic energy of the short, random, collision-time-scale motion of individual grains. Clearly, an equivalent grain temperature could be defined, based on (23). Note too that we obtain the same scaling, $\beta_e^{-1} \sim A^2\omega^2$, for Hamiltonians that incorporate grain rotational energy.

Next, assume that the grain-scale transverse stress autocorrelation function, $\langle \tau_{xy}(t)\, \tau_{xy}(0) \rangle$, decays exponentially, $\langle \tau_{xy}(t)\, \tau_{xy}(0) \rangle = \langle \tau_{xy}(0)\, \tau_{xy}(0) \rangle \exp -t/\tau_c$, and assume that the time constant, τ_c, is on the order of the grain collision time scale, which in turn corresponds to the inverse vibration frequency, $\tau = f_o^{-1} = 2\pi\omega^{-1}$. Noting that $\langle \tau_{xy}(0)\, \tau_{xy}(0) \rangle \sim Nm_g^2A^4\omega^4$, that $V = Nm_g\rho_e^{-1}$, where ρ_e is the effective density of N grains, each of mass m_g, occupying volume V, and carrying out the integral, finally leads to

$$\nu_e \sim 2\pi A^2\omega \tag{24}$$

where $\nu_e = \mu_e\rho_e^{-1}$ is the effective momentum diffusivity, i.e., the kinematic viscosity.

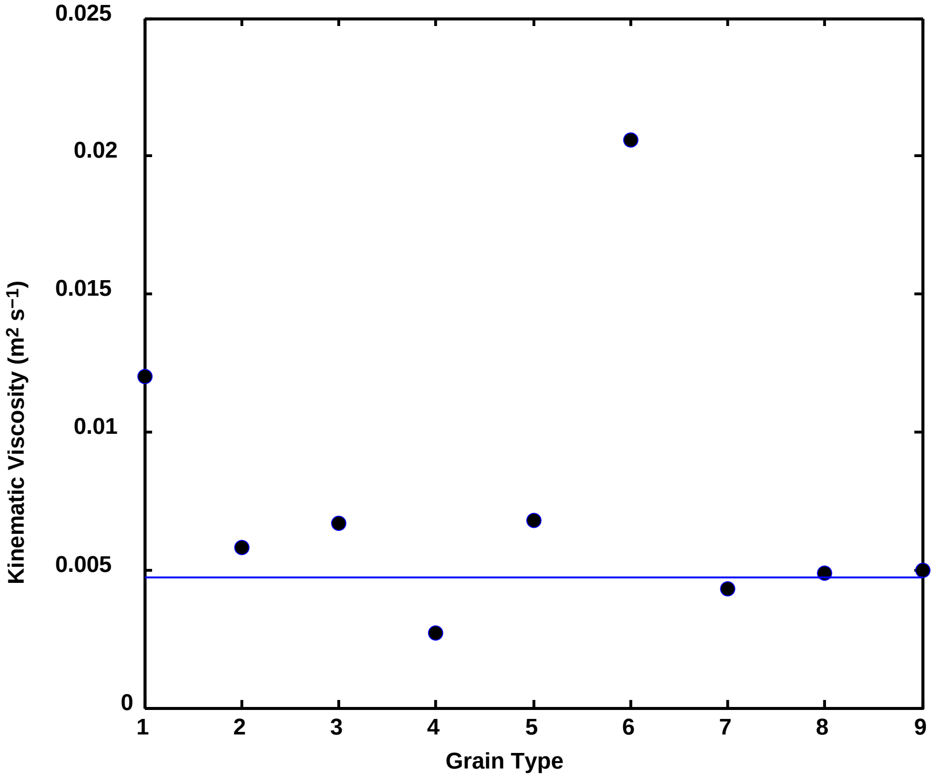

Figure 1: **Comparison of measured kinematic viscosities, ν_e, against approximate grain-independent $\nu_e's$ obtained by scaling.** The grain types, having properties listed in Supplement 3, are: 1) 2050DZ, 2) RCP0909, 3) RS19K, 4) H1008D, 5) RS1022ZS, 6) RS3515DZS, 7) RSG1010, 8) RS1010, 9) mixed media. The relatively large $\nu_e's$ for 2050DZ and RS3515DZS likely reflect the fact that these are approximately 20-30 times more voluminous than the other grain types tested. The rough estimate for ν_e, Eq. (24), obtained by scaling the Kubo relation, Eq. (21), is drawn as a horizontal blue line.

Three observations follow from Eq. (24). First, the estimate suggests that ν_e is largely insensitive to grain shape, as well as grain effective density. Similarly, and consistent with the scaling estimates in Supplement 1 - showing the dominance of grain inertia over elastic and friction forces - the

estimate indicates that ν_e is also insensitive to grain elastic properties. Third, Eq. (24) indicates that ν_e grows quadratically with vibration amplitude and linearly with frequency. Physically, while this remains untested, it suggests a shear thickening effect with increasing vibration amplitude and/or frequency.

In order to test the rough estimate in (24), we compare it against experimentally measured kinematic viscosities, obtained for grain piles comprised of one of nine different grain shapes, each shape composed of either high density plastic, ceramic, or high density ceramic.[11] These experiments, to be reported in a another paper,[11] directly measure the total, time averaged drag force produced on an instrumented cylinder, submerged in vibrating grain piles; see[11] for details.

As shown in Fig. 1, the estimated $\nu_e \sim 4.7(10^{-3})$ m^{2}s^{-1}, corresponding to our experimental conditions, $A = 2(10^{-3})$ m and $\omega = 188.5$ s^{-1}, is qualitatively consistent with the effective $\nu's$ we have observed.

Consistency check 2: Continuum grain flow model and boundary conditions at smooth, vibrating walls

This section serves two purposes. Fundamentally, we want to test, in a simple, preliminary way, the ability of the NS equations to capture the non-trivial toroidal flow pattern we observe via PIV. Practically, we wish to propose a boundary condition appropriate to vibration-driven grain flows in smooth-walled containers.

Flow model

Consider the steady, time average flow of a Newtonian, incompressible fluid in an open hemisphere, where the hemisphere is subject to three modes of vibration, mimicking those in our experiments: i) circular vibration in a vertical plane, ii) precessive rotation about a fixed vertical axis, and iii) azimuthal back-and-forth vibration. In a non- inertial reference frame fixed to the bowl, the non-dimensional, constant viscosity NS equations, stated in terms of vorticity, ω, take the form:

$$Re \frac{D\omega}{Dt} = Re\,(\omega \cdot \nabla)\,\mathbf{v} + \nabla^2 \omega \tag{25}$$

where $Re = v_s R/\nu$ is the Reynolds number, $\mathbf{v}$ is the local grain fluid velocity, $v_s \approx 2\,(10^{-2})\ \mathrm{ms}^{-1}$, is the observed long-time scale average velocity (as measured by PIV or direct observation), $\nu \approx 4\,(10^{-3})\ \mathrm{m}^2\mathrm{s}^{-1}$, is the characteristic kinematic viscosity, as measured in[11] and as estimated above, $R \approx 10^{-1}$ m, is the hemisphere/bowl radius, and D/Dt is the material derivative operator.

With regard to an assumed constant dynamic viscosity, μ,[1,4,5] provide strong evidence that effective grain viscosities are not fixed, but are proportional to the local pressure, P, and inversely proportional to the determinant, $|\dot{\gamma}|$, of the local strain rate tensor. Scaling shows that in our experimental grain piles, pressure varies essentially hydrostatically, and that the characteristic top-to-bottom pressure variation across the grain pile (which includes an inertial $A\omega_o^2$ contribution to the gravitational body force) is only on the order of the 5 % of the background atmospheric pressure. Similarly, scaling indicates that, due to the geometric simplicity and the low Reynolds number character of our experimental flows, spatial variations in strain rate are relatively minor.

Thus, for purposes of preliminary modeling, a fixed viscosity is a reasonable approximation. With regard to density variations, a simple scaling argument shows that the relative density variation, $\Delta\rho/\rho_o$, is on the order of $A/d_{g,e} << 1$, where ρ_o is the measured (bulk) grain density under static conditions, and again, A and $d_{g,e}$ are respectively the vibration amplitude and effective grain diameter. Thus, in our system, the long-time-scale grain flow is effectively incompressible. Finally, while $Re = O\left(0.5\right)$, for simplicity, we neglect the two inertial terms in Eq. (25).

Vorticity boundary condition

The walls of the vibrational bowl in our experiments are smooth, and in flow field measurements about fixed smooth plates, we observe almost pure slip flow, with essentially no sticking. Focusing for illustration on the boundary condition associated with precessive bowl rotation, and referring to Fig. 2, we first set up the kinematics of the time-averaged grain-bowl wall interaction: i) picture the at-wall motion of an idealized, spherical, representative grain (RG), produced by wall precession, where, for small amplitude vibration, the diameter of the RG is significantly larger than A; ii) over one vibration cycle, the RG traces out a bowl-grain interaction envelope, while a fixed point on the wall traces out a (much) smaller circle of radius r' (where the latter can be readily determined as a function of vibration amplitude, A, and position, $\left(\hat{r}, \hat{\theta}\right)$, on the bowl wall); iii) fill the latter circle with a virtual solid disk of grain material, i.e., recognize that over time scales long relative to f_o^{-1}, the circle is, on average, occupied by grains (colliding randomly with the wall); and iv) on the same long time scale, assume that the virtual grain disk rotates at the precessive frequency, f_o.

Next, focus on the long-time-scale dynamics of the virtual disk and assume that the total, time-averaged rotational inertia of the virtual disk (produced by random grain-bowl collisions), equals

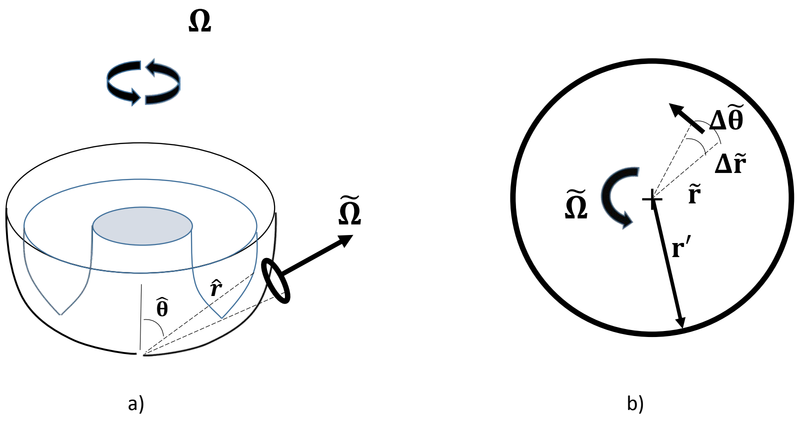

Figure 2: **Derivation of vorticity boundary condition.** For clarity, we show position-dependent grain vorticity generation at the bowl wall produced by precession of the bowl's otherwise vertical rotation vector, Ω. The precessing vector produces position-dependent rotation, $\tilde{\Omega}$, of the bowl; over a large number of bowl rotations, the latter produces a time average, virtual, solid disk of grain material at the wall, depicted as the small tilted disk in **a** and as a circle -viewed in the direction opposite $\tilde{\Omega}$ - in **b.** [Note: $\tilde{\Omega}$ corresponds to Ω_p in Fig. 6.] See text for derivation details.

the total, time- averaged viscous torque produced by grains outside the virtual disk:

$$\frac{1}{\tilde{T}}\left[\int_0^{r'\left(\hat{r},\hat{\theta}\right)} \tilde{r}\Delta m \frac{v^2\left(\tilde{r}\right)}{\tilde{r}} dt'\right] = r'\left(\hat{r},\hat{\theta}\right)\tau\left(r'\right) 2\pi r'\left(\hat{r},\hat{\theta}\right)\ d_g \tag{26}$$

where $\tilde{T} >> f_o^{-1}$ is the averaging period, $\Delta m = \rho_o \tilde{r}\Delta\tilde{r}\Delta\tilde{\theta} d_g$, $v^2\left(\tilde{r}\right)\tilde{r}^{-1} = \Omega_o^2\tilde{r}$, $r' = r'\left(\hat{r},\hat{\theta}\right)$, and $\Omega_o = 2\pi f_o$. The viscous shear at the edge of the virtual disk is $\tau\left(r'\right) = \mu_e \frac{\partial v_\psi}{\partial n} = \mu_e \omega_t\left(\hat{r},\hat{\theta}\right)$, where $\omega_t\left(\hat{r},\hat{\theta}\right)$, is the local vector vorticity produced by precession. [The partial derivative represents the (local) change in the grain velocity component, v_ψ, parallel to $\tilde{\Omega}$, in the local wall normal direction.]

A similar approach can be used to derive time-averaged, long-time-scale vorticity produced by

the other two components of bowl vibration. Given these boundary conditions, the steady state vorticity equation governing the time-averaged vorticity field can be solved analytically, allowing, in turn calculation, via the Biot-Savart relation, the steady time-averaged velocity field. Further details will be reported in a forthcoming paper.

As shown in Fig. 6, this relatively simple model captures the non-trivial helical grain flow that we observe. Importantly, and consistent with observed short-time-scale, single grain dynamics - Fig. 3, and long-time- scale collective grain hydrodynamics - Fig. 4, we observe another significant signature - the steady (time-averaged) flow of a vibration- driven atomic liquid/dense gas in a vibrated hemisphere - of molecular hydrodynamics in vibrated grain piles.

References

[1] Forterre, Y. & Pouliquen, O. Flows of Dense Granular Media. *Annu. Rev. Fluid Mech.* **40,** 1-24 (2008).

[2] Haff, P. K. Grain flow as a fluid-mechanical phenomenon. *J. Fluid Mech.* **134,** 401-430 (1983).

[3] Bougie, J., Kreft, J. B. & Swinney H. L. Onset of Patterns in an Oscillated Granular Layer: Continuum and Molecular Dynamics Simulaations. *Phys. Rev. E* **71,** 021301 (2005).

[4] Arenson, I. S. & Tsimring, L.S. Patterns and collective behavior in graanular media: Theoretical concepts. *Rev. Mod. Phys.* **78,** 641-692 (2006).

[5] Govender, I. Granular flows in rotating drums: A rheological perspective. *Mineral Engrg.* **92,** 168-175 (2016).

[6] Hung, C.-Y., Stark, C. P. & Capart, H. Granular flow regimes in rotating drumsfrom depth-integrated theory. *Phys. Rev. E* **93,** 030902(R) (2016).

[7] Kadanoff, L. P. & Martin, P. C. Hydrodynamic Equations and Correlation Functions. *Annals Phys.* **24,** 419-469 (1963).

[8] Forster, D. *Hydrodynamic Fluctuations, Broken Symmetry, and Correlation Functions.* Perseus, New York (1990).

[9] Boon, J. P. & Yip, S. *Molecular Hydrodynamics.* McGraw-Hill, New York (1980).

[10] Kubo, R. Statistical-Mechanical Theory of Irreversible Processes. I. *J. Phys. Soc. Japan* **12,** 570-586 (1957).

[11] Fleischhauer, E., Keanini, R. G., Mullany, B. & Tkacik, P. Experimental Measuremnement of Effective Viscosities. To be submitted *J. App. Phys.* (2016). (Reference available to referees on request.)

[12] Evans, D. J. & Morriss, G. P. *Statistical Mechanics of Nonequilibrium Liquids.* ANU E Press, Canberra (2007).

[13] Gardiner, C. W. *Handbook of Stochastic Methods, 3rd Ed.* Springer-Verlag, Berlin (2004).

[14] Mori, H. Transport, Collective Motion, and Brownian Motion. *Prog. Theor. Phys.* **33,** 423-455 (1965).

[15] Zwanzig, R. Time-Correlation Functions and Transport Coefficients in Statistical Mechanics. *Rev. Phys. Chem.* **16,** 67-102 (1965).

[16] Pathria, R. K. & Beale, P. D. *Statistical Mechanics, 3rd Ed.* Elsevier, Oxford (2011).

Supplement 3: Grain properties

R. G. Keanini, Peter T. Tkacik, Eric Fleischauer,

Hossein Shahinian, Jodie Sholar, Farzad Azimi and Brid Mullany

Department of Mechanical Engineering and Engineering Science

The University of North Carolina at Charlotte

Charlotte, North Carolina 28223-0001

**Name**	**Image**	**Material**	**Nominal Dimensions (mm)**	**Density $(\mathbf{kg\,m^{-3}})$**
2050DZ	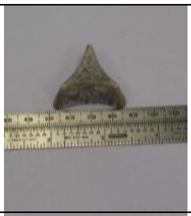	Plastic	36 x 13	752
RCP0909	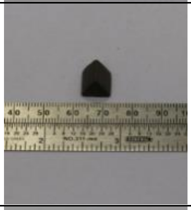	Ceramic	9 x 9	2033
RS19K	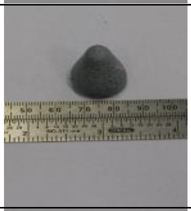	Ceramic	19 x 20	1711
H1008D	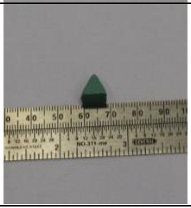	Plastic	9 x 6 x 8	2033

RS1022ZS	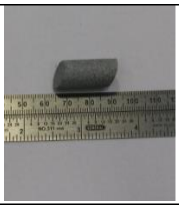	Ceramic	10 x 20	1600
RS3515DZ S	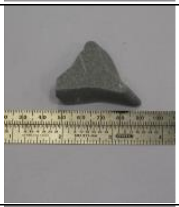	Ceramic	35 x 15	1285
RSG1010	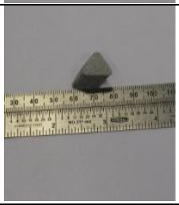	Ceramic	10 x 10	1390
RS1010	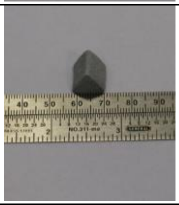	Ceramic	10 x 10	1147
Mixed Media	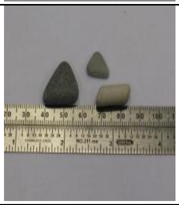	Ceramic and Plastic	5 x 5 to 10 x 15	1615

Figure 1: Grain properties. Dimensions represent maximum lengths of single grains in mutually perpendicular directions. Measured densities are those of the solid grain material. For H1008D, there is a slight taper in the (triangular) cross-sectional area of the grain: over the 6 mm height of the grain, the side length of the equilateral triangle decreases from 9 to 8 mm. For mixed media, grain sizes fall within the dimensions shown.
